# An online experimental medicine trial on the effect of 28-day simvastatin administration on emotional processing, reward learning, working memory and salivary cortisol in healthy volunteers at risk for depression: OxSTEP protocol

**DOI:** 10.1192/bjo.2023.44

**Published:** 2023-06-14

**Authors:** Shona Waters, Riccardo De Giorgi, Alice M. G. Quinton, Amy L. Gillespie, Susannah E. Murphy, Philip J. Cowen, Catherine J. Harmer

**Affiliations:** Department of Psychiatry, University of Oxford, UK; Department of Psychiatry, University of Oxford, UK; and Warneford Hospital, Oxford Health NHS Foundation Trust, UK

**Keywords:** Simvastatin, emotional processing, online experimental medicine study, loneliness, depression

## Abstract

**Background:**

Evidence suggests inflammation may be a key mechanism by which psychosocial stress, including loneliness, predisposes to depression. Observational and clinical studies have suggested simvastatin, with its anti-inflammatory properties, may have a potential use in the treatment of depression. Previous experimental medicine trials investigating 7-day use of statins showed conflicting results, with simvastatin displaying a more positive effect on emotional processing compared with atorvastatin. It is possible that statins require longer administration in predisposed individuals before showing the expected positive effects on emotional processing.

**Aims:**

Here, we aim to test the neuropsychological effects of 28-day simvastatin administration versus placebo, in healthy volunteers at risk for depression owing to loneliness.

**Method:**

This is a remote experimental medicine study. One hundred participants across the UK will be recruited and randomised to either 28-day 20 mg simvastatin or placebo in a double-blind fashion. Before and after administration, participants will complete an online testing session involving tasks of emotional processing and reward learning, processes related to vulnerability to depression. Working memory will also be assessed and waking salivary cortisol samples will be collected. The primary outcome will be accuracy in identifying emotions in a facial expression recognition task, comparing the two groups across time.

Depression is common and associated with considerable health disability. Around a third of patients are resistant to current treatments.^1^ Thus, there is a pressing need to develop antidepressant medications with novel mechanisms of action or, conversely, to identify alternative pathophysiological pathways leading to depression that can be targeted with new treatments.^2^ A validated method to investigate this area is based on the cognitive neuropsychological model of depression.^3^ Both people with depression and those at risk for developing depression consistently show negative biases in emotional processing.^4–6^ This bias has been demonstrated reliably in tasks of facial expression recognition, memory and attention,^7–9^ and is thought to play a key role in the development and maintenance of clinical depressive symptoms.^10^ Acute antidepressant administration has been found to reduce these negative biases of emotional information in healthy controls and patients with depression.^11–13^ Further, early changes in these biases are associated with later antidepressant response.^14,15^ Reward learning has also been reported to be affected by depression and anhedonia, with depression found to be associated with both reduced sensitivity to reward and heightened sensitivity to loss.^16,17^ Antidepressants have been associated with enhanced reward learning,^18^ and specifically, reduced sensitivity to loss.^19^ Non-emotionally salient cognition such as working memory is also an associated impairment of depression,^10^ but, unlike emotional cognition, evidence that conventional antidepressants improve this symptom is conflicting.^20^

## Neuroinflammatory hypothesis of depression

Both emotional biases and impaired cognitive function have been associated with inflammation. For example, treatment with the proinflammatory cytokine interferon-α has been reported to induce a negative emotional bias, reduce motivation and increase anhedonia,^21,22^ and the induction of inflammation has been associated with working memory deficits in preclinical^23^ and human^24,25^ models.

There is a growing body of evidence supporting a neuroinflammatory hypothesis of depression.^26^ Raised inflammation and hypothalamic-pituitary-adrenal (HPA) axis hyperactivity are often associated, and are two of the most consistent biological findings in patients with depression.^27^ Multiple studies have shown that manipulating inflammatory pathways induces depression.^28^ HPA axis dysfunction and an elevated cortisol response to stress have also been associated with depression.^29^

Overall, an experimental medicine approach that explores the effects of medications with anti-inflammatory potential on measures of emotional processing, reward learning and working memory can provide valuable information before full-scale, randomised controlled trials to assess efficacy.

## Statins and neuropsychiatry

In recent years, there has been a significant interest in the investigation of the effects of statins in neuropsychiatric disorders, and especially depression.^30^ Statins are a class of medications usually prescribed to reduce peripheral cholesterol by inhibiting the liver enzyme 3-hydroxy-3-methylglutaryl coenzyme A reductase, with subsequent beneficial cardiometabolic effects.^31^ In addition, statins have widespread influences on neurobiological and immune systems and inflammatory pathways involved in depression pathophysiology.^32^ There are several examples of biological mechanisms that could support the antidepressant potential of statins.^32^ These include reduction of microglial and astrocyte activation, and inhibition of central cytokine release.^33–35^ Alternatively, *in vitro* studies have suggested 5-hydroxytryptamine 1a receptor dynamics are altered by statin-medicated cholesterol depletion.^36^ Because of their established safety profile, statins are ideal candidates for repurposing in the treatment of depression. However, inconsistencies between preclinical^37,38^ and clinical^37,39–43^ evidence mean that their role in the management of depressive disorders remains unclear. Such inconsistencies may be explained by differing study designs, including heterogeneous populations, interventions/exposures and outcomes of interest.^44^

## Loneliness, social isolation and the COVID-19 pandemic

It has been hypothesised that a raised baseline inflammatory load, in relation to risk factors such as age, comorbidities and adverse life events, could make certain people more likely to benefit from the anti-inflammatory and thus putative antidepressant effects of statins.^37^ In this context, the COVID-19 pandemic and its potential consequences on mental health^45^ highlight the need to be able to reduce the risk of developing depression fast and at scale. Social isolation and loneliness, both exacerbated by the measures needed to control the COVID-19 pandemic, have been associated with vulnerability to depression,^46^ possibly via a causal mechanism involving inflammation.^47,48^ The identification of strategies that may be protective against the negative mental health consequences of such psychosocial stressors would therefore have relevance on a large population scale.

An observational study carried out during the COVID-19 pandemic highlighted that, compared with several other medications (including antihypertensives), statin use was associated with less negative bias in tasks of emotional processing and lower sensitivity to loss in a reward processing task.^49^ These findings suggest that reducing inflammation via a statin could have a protective effect against depression in the context of the COVID-19 pandemic and its aftermath, at times of high levels of psychosocial stress such as social isolation and loneliness. We recently conducted a study in healthy participants showing that 7-day atorvastatin, compared with placebo, worsened emotional negative bias by increasing the identification of fearful facial expressions; this occurred independently from subjective states of mood, anxiety and peripheral markers of inflammation.^50^ Seven-day simvastatin, on the other hand, was associated with some evidence of improvement in negative bias (i.e. more positively valenced intrusions in emotional recall), but also with an increase in subjective scores of anxiety compared with controls.^51^ Compared with atorvastatin, simvastatin is a more lipophilic molecule, and is therefore more capable of crossing the blood–brain barrier and potentially expressing an effect in the central nervous system.^52^ Clinical evidence suggests a stronger antidepressant potential of the more lipophilic simvastatin compared with atorvastatin.^39,41,53^ Neither study found any effect of statins on reward learning or non-emotional cognition.^50,51^ Findings from these latter experimental medicine trials^50,51^ seem to differ from the earlier observational study.^49^ Moreover, they do not appear in keeping with the possible antidepressant effect of statins seen in clinical populations.^37^ A possible explanation is that, although changes in emotional processing mechanisms can be seen after 7 days, the intricate pharmacological properties of statins^32^ means that they may require longer administration in predisposed (i.e. at risk for depression) individuals before showing the expected positive effects on emotional processing and reward learning,^49–51^ and, following that, on mood and anxiety.^37^

To verify this hypothesis, we designed the current experimental medicine trial: the Oxford Study of Simvastatin and Emotional Processing (OxSTEP). This study will assess the effects of 28-day simvastatin administration versus placebo on emotional processing, reward learning, working memory and waking salivary cortisol in people who are at risk for depression because of high levels of loneliness.

## Objectives and hypothesis

### Primary objective

The primary aim of this study is to test the neuropsychological effects of statins by assessing the effect of 28-day administration of statin treatment on emotional processing, reward learning and working memory compared with placebo, in healthy volunteers at risk for depression owing to loneliness.

### Secondary objectives

Our secondary objectives include using waking salivary cortisol as an index of HPA axis activation, indicating biological stress and arousal; collecting mood and anxiety questionnaires to examine if any underlying changes in emotional processing correlates with subjective measures; and looking at the effect of statin administration within specific subgroups.

Our prediction is that, relative to placebo, in this group of participants at risk for depression because of high levels of loneliness, 28-day simvastatin use will lead to positive effects on emotional processing (decreased negative bias), reward learning (increased sensitivity to reward versus loss) and working memory (increased accuracy on the N-back test) compared with placebo, as well as lowering waking salivary cortisol concentrations.

## Method

### Study setting

OxSTEP is an online, double-blind, parallel-group, randomised, gender-stratified, placebo-controlled experimental medicine trial. One hundred participants from across the UK will be recruited through online advertising. Researchers are based in the Department of Psychiatry at the University of Oxford. Participant enrolment started in July 2021 and is expected to end in March 2023. All data remains blinded at the point of submission.

### Sample size calculation

The sample size calculation was based on our primary outcome of accuracy on the facial expression recognition task, part of the Oxford Emotional Test Battery, a validated tool to assess emotional processing.^11,54,55^ We initially computed that a sample size of 25 per study arm would give 0.9 power to detect changes of the magnitude of those we have seen in a previous key study (drug mean 10.64 (s.d. 9.77) versus placebo mean 3.36 (s.d. 5.96)^12^). However, it is possible that online testing will have a reduced sensitivity to change in emotional processing compared with face-to-face testing in a laboratory. Additionally, a smaller magnitude of change would still be a clinically important difference that we would want to detect. For this reason, we have planned to recruit 50 participants per arm; this is made possible by the increased efficiency of online testing in participant recruitment.

### Recruitment

After responding to an online advert, potential participants will be shown the Participant Information Sheet and asked to complete a short online pre-screening form, comprising a brief questionnaire referring to the main inclusion/exclusion criteria, including the UCLA 3-item Loneliness Scale^56^ to assess for baseline loneliness level. Those who preliminarily appear to meet the inclusion criteria will be invited to a pre-screening telephone call with a researcher, where key criteria will be confirmed and informed consent explained. After participants have given online informed consent, they will have a screening session via video call with a study psychiatrist. During the screening, consent will be re-checked and information will be taken about medical history, concomitant medication, psychiatric history and current psychiatric symptoms using the Structured Clinical Interview for DSM-5 (SCID-5).^57^ Inclusion and exclusion criteria are reported in Table 1. A score of 6 or more on the UCLA 3-item Loneliness Scale indicates moderate to severe loneliness, and is a conservative assessment of the risk of depression.^58^ The three-item scale correlates well with the more comprehensive Revised UCLA Loneliness Scale.^56,59^ Other eligibility criteria have been chosen to (a) recruit a healthy adult population, for whom it would be safe to take the medication without significant clinical oversight; and (b) limit extreme cognitive and clinical heterogeneity.

**Table 1:** Participant eligibility criteria

Participant eligibility criteria
Inclusion criteria
Male or female
Aged 21–65 years
At risk for depression as measured by a score ≥6 on the UCLA 3-item Loneliness Scale
Body mass index in the range of 18–30 kg/m^2^
Willing and able to give informed consent for participation in the study
Registered with a general practice and consent to their general practitioner being informed of participation in the study
Currently living in the UK and sufficiently fluent in English to understand and complete the tasks
Able to access and use a computer with internet
Able to complete online questionnaires and tasks
Exclusion criteria
Currently on any regular prescribed medications (except the contraceptive pill), unless unlikely to compromise safety or affect data quality in the opinion of the investigator
History or current significant psychiatric illness (other than past (>6 months) episodes of depression or anxiety)
Current alcohol or substance misuse disorder (<6 months)
History of, or current significant hepatic disease
History of, or current significant neurological condition (e.g. epilepsy)
History of haemorrhagic stroke or deep brain structure stroke
Known hyperglycaemia/prediabetes/diabetes
Known hypersensitivity to the study drug (i.e. simvastatin) or sucrose
Pregnant, breast feeding or women of child-bearing potential who are not using appropriate contraceptive measures
Participation in a study that uses the same or similar computer tasks (apart from the N-back task) as those used in the present study
Participation in a study that involves the use of a medication within the past 3 months

We aim to minimise the number of participants lost to follow-up by respecting participants commitments outside of the study and working flexibly around them, regularly checking in with participants to keep them on track and engaged. Such flexibility is made possible by the innovative remote design of this study. Participants will be given a contact number and advised to get in contact if they have any concerns or their health status changes. If necessary, the researcher will contact the study medic. A participant will be discontinued from the study and advised to stop taking the study medication if deemed necessary by the medical lead. Withdrawn participants will be replaced and their data will not be included in the analysis.

### Randomisation

Using online randomisation tool Sealed Envelope version 1 (Sealed Envelope Ltd, London, UK; https://www.sealedenvelope.com/simple-randomiser/v1/lists), an uninvolved researcher will generate a randomisation code in blocks of four, stratified by gender, which will be stored in a sealed envelope in a lockable cabinet. Eligible participants will be randomised to receive either simvastatin 20 mg (50 participants) or placebo (50 participants). A dose of 20 mg is safe^60^ and has been successfully used in trials on depression in the past.^61^ Randomisation will occur up to a maximum of 4 weeks after screening, by one of the study researchers. Participants will be posted 30 days of the allocated drug/placebo to their address, as well as full written instructions of how and when to take them, and secure pre-paid postal boxes to return their saliva samples. Both the participants and the study researchers will be blinded to the study medication.

### Procedures

**Fig. 1:**
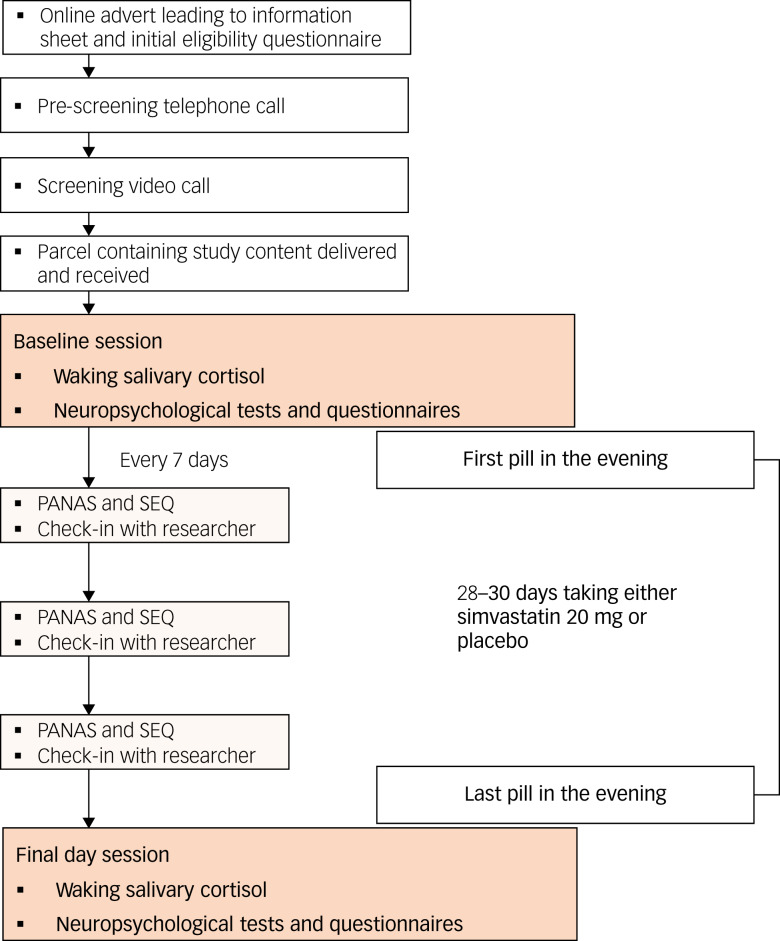
Flow chart summarising the study design. PANAS, Positive and Negative Affect Schedule; SEQ, Side-Effects Questionnaire.

Enrolled participants will be instructed to contact the study researchers when they receive their parcels. At this juncture, a researcher will call them to once again go through the study procedure and to schedule the study dates. Participants must complete the baseline session before the first dose of their allocated medication. Participants will complete a remote baseline session, followed by 28–30 days of study medication, followed by a remote final day session (see Fig. 1). This is specifically designed to enable participants to schedule both sessions around their own commitments.

### Baseline procedures

On their first day, participants will take waking cortisol saliva samples – four samples taken 15 min apart, as per standard operating procedure. Each sample is collected with a cotton swab and salivette,^62^ pre-labelled with the anonymised subject identifier and marked 1 to 4. Participants are instructed to take the samples according to this order, remaining in bed with the lights off and without food or drink. They are asked to mark the time of collection on each tube, before returning them by post in the secure pre-paid box. Participants will then complete an online baseline assessment (approximately 1.5 h) via the GDPR-compliant online software platform Gorilla Experiment Builder (Cauldron Science Ltd, Newbury, UK; www.gorilla.sc). This will involve a battery of established neuropsychological tasks (see below), including the Emotional Test Battery (ETB) for emotional processing, the Probabilistic Instrumental Learning Task (PILT) for reward learning and the N-back task for working memory. Participants will go on to a battery of questionnaires, including a COVID-19-related questionnaire, the Centre for Epidemiologic Studies Depression Scale,^63^ Positive and Negative Affect Schedule (PANAS),^64^ Snaith–Hamilton Pleasure Scale,^65^ State–Trait Anxiety Inventory,^66^ Perceived Deficit Questionnaire^67^ and a side-effects questionnaire (SEQ).

Participants will begin taking the study medication on the evening of their baseline assessment, and continue for 28–30 days.

### Subsequent procedures

During the 28–30 days of medication administration, participants will be asked to complete weekly SEQ and PANAS questionnaires. A researcher will also contact the participant weekly to check there are no concerns and confirm they are concordant with the study protocol. To improve medication adherence, participants will receive daily automated reminder text messages via FireText (FireText Communications Ltd, Penryn, UK; www.firetext.co.uk).^68^

The online research session will take place after 28–30 days of simvastatin/placebo administration. On the morning of the final day session, participants will take another waking saliva sample. They will also repeat the questionnaires and complete a second version of the neuropsychological tasks. Finally, to measure success of blinding, participants will be asked to guess whether they were taking simvastatin or placebo. A researcher will contact the participants on their first and last day visits, ensuring the individual successfully took and posted the saliva samples and the online tasks are completed.

Saliva samples, which arrive by post, will be processed and stored as per standard operating procedure, by a trained researcher. Samples will be centrifuged at 1000 g for 2 min before being transferred into labelled 5 mL polypropylene tubes under a Class 2 biohazard hood. Tubes will be placed in a storage box within a clinical freezer at −20°C. This has been tested and validated as a reliable method of cortisol measurement.^69–71^

### Neuropsychological tasks

#### ETB

We will assess emotional processing via three validated computerised tasks from the ETB:^11^ the facial expression recognition task (FERT), the emotional categorisation task (ECAT) and the emotional recall task (EREC). These have been described in full elsewhere.^50^

In the FERT, facial expressions of six emotions (anger, disgust, fear, happy, sad and surprise) and a neutral expression are randomly displayed on the screen for 500 ms. Participants must respond by identifying the expression as quickly and accurately as possible. Facial expressions are adapted from the Karolinska Directed Emotional Faces set,^72^ and are depicted at a range of intensity levels.^73^ The primary outcome will be accuracy at identifying the correct emotion. Additional outcomes will be misclassifications and mean reaction times. To assess discriminability (d’, a measure of sensitivity) and response bias (β, a measure of conservativeness),^74^ a signal detection analysis will be carried out. Unbiased hit rate will be used to measure accuracy while considering response bias.

In the ECAT, 20 positively and 20 negatively valenced personality characteristic words^75^ are randomly displayed on the computer screen for 500 ms. Participants must indicate, as quickly and accurately as possible, whether they would ‘like’ or ‘dislike’ to be described by this word. Accuracy, mean reaction time, discriminability and response bias will be assessed. After a delay of around 10–20 min, participants carry out the EREC, a free-recall task. Here, participants are given 4 min to type the personality characteristic words they remember from the ECAT. The number of correctly and falsely recalled positive and negative words will be assessed.

#### PILT

We will assess reward learning via the PILT (adapted from Pessiglione et al^76^). In this task, the aim is to win as much money as possible by picking between two symbols that are displayed on the computer screen for 4000 ms. Participants begin with £1. Across three blocks, there are 90 win trials, where one symbol of each pair will result in winning £0.20 and the other in no change; and 90 loss trials, where one symbol of each pair will result in no change and the other in losing £0.20. For both win and loss trials, one symbol will result in the better outcome 70% of the time, and the other 30% of the time. Feedback on the outcome is given after each trial. Participants have to use this to learn over time which symbols are associated with high probability to win, and which are associated with high probability to lose. Probability of choosing the winning symbol in win trials and the losing symbol in loss trials will be assessed, as well as end total money, amount won and lost, and proportion of trials where the participant has switched symbol within the same condition.

#### N-back task

The N-back task assesses working memory.^77^ Participants respond to whether letters, appearing sequentially on the screen, match the letter presented N-trials before. Four conditions will be used: 0-back, where participants respond by pressing the ‘m’ or ‘n’ key (yes or no, respectively) if the letter presented is an ‘X’ or not. Followed by one-back, two-back and three-back conditions, asking whether the letter is the same as it was one, two and three trials ago, respectively. This task employs a block design, where each condition has two blocks of 20 trials. Accuracy and mean response times for correct trials for each condition will be assessed, as well as discriminability and response bias.

#### Analysis

Demographic and baseline measures will be reported descriptively. Loneliness, mood, anxiety, side-effects and salivary cortisol measures will be analysed using repeated measures analysis of variance (ANOVA), with group (simvastatin versus placebo) as the between-participants factor and time (baseline versus final day session) as the within-participant factor. Feasibility outcomes for the remote design will be reported, such as drop-out rate, within-study exclusions, adherence and missing data rates.

Data distributions will be visually checked for all neuropsychological tasks, using boxplots. Extreme outliers (i.e. data values lying more than three times outside the interquartile range) will be excluded. The resulting data will be analysed with ANOVA, with group as the between-participants factor and time as a within-participant factor. The individual tasks will be analysed with the following additional within-participant factors: for FERT, emotion; for ECAT and EREC, valence; for PILT, win or loss; and for N-back, trial condition.

On the basis of potentially diverse pathophysiological effects of simvastatin, we will further explore the main outcomes (FERT accuracy, misclassifications, reaction times) in specific subgroups of participants (females versus males, age 21–40 *v.* 41–65 years, body mass index 18–25 kg/m^2^ (normal weight) *v.* 26–30 kg/m^2^ (overweight), self-report of family history of mental disorder positive versus negative). These subgroups will be added as between-participants variables in our ANOVAs. *Post hoc* sensitivity analysis to account for oral contraceptive pill use will be carried out.

Any significant interactions will be followed up using simple main effect analyses. When assumptions of equality of variances are not fulfilled, the Greenhouse–Geisser procedure^78^ will be used to correct the degrees of freedom.

### Patient and public involvement

A remote focus group was held with members of the Oxford Biomedical Research Centre patient and public involvement contributor pool during the development of the study design. Discussions focused on their perspectives regarding use of statins in a psychiatric context (in particular, as a preventative intervention), and appropriate inclusion criteria for the study. Patient and public involvement contributors highlighted the inclusivity an online study design would generate, with increased accessibility for participants who usually do not have opportunities to participate in research. A key outcome of the consultation was that the study should target people at risk of depression, as opposed to generally healthy volunteers, to better justify taking statin medication for a prolonged period of time and to explore a preventative context.

### Ethics

The study researchers will take responsibility for the conduct of OxSTEP, supervising the operation of the project on a day-to-day basis, and ensuring good clinical practice guidelines are followed at all times. OxSTEP team members will monitor the data at the University of Oxford, keeping procedures aligned with the study protocol and ensuring proper study management and completion of study procedures in a timely manner.

Data will be stored on institution drives and cloud systems, with security measures in place. Hard-copy files will be stored in a secure location in a locked cabinet. Study data will be de-identified and stored separately from a linking log. Only study researchers will have access to study data.

The authors assert that all procedures contributing to this work comply with the ethical standards of the relevant national and institutional committees on human experimentation and with the Helsinki Declaration of 1975, as revised in 2008. All procedures involving human participants were approved by the University of Oxford Central University Research Ethics Committee (approval number MS-IDREC R73946/RE001). The study protocol is registered on Clinicaltrials.gov (identifier NCT04973800). Any updates to the protocol will be registered as an amendment through the ethics committee and then amended on Clinicaltrials.gov.

## Discussion

There is conflicting evidence regarding the association between statins and depression.^37,39–42^ Although there are strong associations between inflammation, emotional and reward processing, and depression, the mechanisms remain poorly understood. Recent evidence seems to suggest a mechanism by which loneliness and social isolation are associated with depression is via inflammatory pathways.^47,48^ Relatedly, recent studies have shown that different subgroups of patients react differently to statin interventions.^44,79^ The development of this online methodology, in combination with the COVID-19 pandemic and the measures used to counter it, have created an opportunity to study a specific group at risk for depression.

### Strengths and limitations

A remote study design in experimental medicine has clear strengths. It allows the recruitment of a diverse study cohort, with populations that may have otherwise not been represented by equivalent in-person research;^80^ in a recent study using these tasks online, the racial diversity of the study population was broadly reflective of the UK population.^49^ Where many studies have been severely disrupted by the COVID-19 pandemic,^81^ our design is robust to changes in government measures and participants or their contacts testing positive for COVID-19. Unnecessary travel by participants to the study site saves time, money and convenience. However, careful consideration needs to be taken to ensure the quality of data collected is to an equal standard as in-person testing. Being outside a controlled laboratory relies on self-reported measures and trust in participants, potentially leading to less reliable results. We aim to minimise this risk by building rapport with participants through regular contact, using engagement checks before each online task, and targeting our advertising to appeal to participants with a genuine interest in helping inform research. Participants known to have deviated from protocol will be excluded. Outliers will be excluded from analysis. Additionally, because of the practical restraints of a fully remote study, participants will not be recruited based on raised inflammation levels and blood immune biomarkers will not be measured. Although this would benefit the clarity of our hypothesis, recruiting participants who score high on loneliness criteria, which is associated with raised inflammation,^47,48^ should be sufficient to study our hypothesis.

In addition to the online element, the study has other strengths. For example, outcomes will be measured before and after intervention, allowing us to measure individual differences that may be relevant for our heterogenous cohort.^82^

In conclusion, this innovative approach to experimental medicine studies in depression, employing a novel, online study design on established neuropsychological tasks, can have an important translational value by shedding some light upon the interactions between statins, HPA axis and several cognitive functions that have been linked to depression. Furthermore, if this new methodology is proved to be feasible and reliable, it could inform further experimental medicine trials.

## Data Availability

The data produced by this study will be available from the corresponding author upon reasonable request, following unblinding, analysis and publication of findings.
